# Novel estrogen-related gene variants identified by whole-exome sequencing in pregnancy-associated intrahepatic cholestasis

**DOI:** 10.3389/fgene.2025.1626890

**Published:** 2025-08-26

**Authors:** Hua Lai, Siming Xin, Jinliang Zhang, Yang Hu, Wenjuan Fan, Hong Wan, Bowen Chen, Yang Zou, Xiaoming Zeng, Xianxian Liu

**Affiliations:** Central Laboratory, Jiangxi Maternal and Child Health Hospital, Nanchang, Jiangxi, China

**Keywords:** intrahepatic cholestasis of pregnancy, whole-exome sequencing, estrogen-related genes, CYP17A1 gene, novel variants

## Abstract

Intrahepatic cholestasis of pregnancy (ICP) is associated with an increased risk of adverse fetal outcomes, including fetal morbidity and mortality. It is a complex liver disorder influenced by genetic interactions, estrogen levels, and environmental factors. Although elevated estrogen levels are known to contribute to ICP pathogenesis, the role of genetic variants in estrogen-related genes remains poorly characterized. Accordingly, we conducted whole-exome sequencing (WES) in 249 patients with ICP, focusing on eight key estrogen-related genes (*ESR1/2, CYP17A1/19A1, CYP1A2/1B1/3A4*, and *COMT*). Variants were validated by Sanger sequencing and functionally characterized using comprehensive bioinformatics analyses (PolyPhen-2, SIFT, and MutationTaster) combined with molecular modeling. Our whole-exome sequencing analysis of 249 patients with ICP identified 235 variants across eight estrogen-related genes, with three novel *CYP17A1* missense mutations (p.Pro28Thr, p.Phe93Leu, and p.Arg347Leu) demonstrating particularly significant findings. These variants exhibited the following characteristics: (1) complete absence in 1,237 controls and all public genomic databases (1000 Genomes, ExAC, and dbSNP); (2) evolutionary conservation of the affected residues, with unanimous pathogenic predictions from all algorithms (PolyPhen-2: damaging; SIFT: deleterious; MutationTaster: disease-causing); (3) molecular modeling demonstrating structural perturbations in critical functional domains, including steroid-binding and redox partner interaction sites. Furthermore, analysis of placental tissue revealed significantly reduced *CYP17A1* expression in ICP cases *versus* controls (*P* < 0.05), suggesting functional impairment of estrogen metabolic pathways. We identified three novel pathogenic *CYP17A1* variants associated with ICP through whole-exome sequencing, elucidated their structural and functional effects on estrogen metabolism, and demonstrated significantly reduced placental *CYP17A1* expression, thereby providing crucial insights into the genetic basis of ICP pathogenesis.

## Introduction

Intrahepatic cholestasis of pregnancy (ICP) is a common gestational complication characterized by pruritus, elevated total serum bile acids (TSBAs), and abnormal alanine aminotransferase (ALT) and aspartate transaminase (AST) levels, usually occurring in the second and third trimesters when the serum estrogen and progesterone levels are highest (Kenyon et al., 2002). ICP symptoms, including pruritus and elevated bile acids/liver enzymes, typically resolve within 4 weeks postpartum as pregnancy hormones normalize (Wikstrom Shemer et al., 2013). The prevalence of ICP has been reported to range from 0.1% to 15.6%, depending on the country and population (Geenes and Williamson, 2009; Lammert et al., 2000). Its prevalence is higher in countries such as Chile and Sweden, along with the Yangtze River valley in China (Mella et al., 1996). The recurrence rate of ICP is as high as 40%–60% (Ovadia and Williamson, 2016).

ICP may predispose mothers to pregnancy complications and postpartum morbidity and increase the risk of perinatal morbidity and mortality. Recent studies have shown that pregnant women with ICP may have a higher risk of developing pre-eclampsia or gestational diabetes (Girling et al., 2022; Martineau et al., 2014; Rezai et al., 2015). Moreover, these women have a significantly increased risk of developing hepatobiliary disorders or metabolic diseases later in life (Marschall et al., 2013). In addition, elevated maternal bile acids pose a significant risk to fetal health, and multiple studies have demonstrated that when maternal TSBA levels reach ≥40 μmol/L, pregnancy faces an increased risk of several complications. These include meconium-stained amniotic fluid, spontaneous preterm labor, low Apgar scores, and fetal asphyxia (Geenes et al., 2014; Glantz et al., 2004; Sarker et al., 2022). A comprehensive meta-analysis of ICP identified 100 μmol/L as the critical threshold for risk of stillbirth (Ovadia et al., 2019). Evidence suggests that the increased risk of stillbirth might be linked to the toxicity of BAs to fetal cardiomyocytes or to the vasoconstriction of chorionic vessels (Miragoli et al., 2011; Sepulveda et al., 1991; Williamson et al., 2001; Williamson et al., 2011). Although ICP is the most prevalent hepatobiliary disorder during pregnancy, effective preventive strategies remain unavailable. Currently, first-line treatment with ursodeoxycholic acid has been shown to relieve pruritus and reduce TSBA levels to some extent; however, it has not been shown to improve adverse perinatal outcomes (Fleminger et al., 2021; Wood et al., 2018). The primary reason for this condition is that people lack a clear understanding of the underlying mechanisms of ICP. Therefore, it is important to further elucidate the etiology and pathophysiology of ICP and its relationship with fetal morbidity.

To date, it is widely accepted that estrogen, genetic predisposition, underlying liver disease, and environmental risk factors primarily contribute to the development and severity of ICP disease (Dixon et al., 2022; Dixon and Williamson, 2016; Turro et al., 2020; Williamson and Geenes, 2014). Evidence from observational studies has suggested that the incidence of ICP is characterized by geographical location, ethnicity, recurrence, familial clustering tendency, and altered environmental factors, such as vitamin D or natural selenium deficiency during the winter (Abedin et al., 1999; Eloranta et al., 2001; Reyes et al., 2000; Reyes et al., 1976; Wikstrom Shemer and Marschall, 2010). Experimental evidence has demonstrated that estrogen compounds, including ethinylestradiol and estradiol benzoate, can successfully reproduce the ICP pathology in rodent models (Schreiber and Simon, 1983; Stieger et al., 2000). Women with a history of oral contraceptive use are more likely to develop ICP (Guth et al., 2016; Kremer et al., 2015). Epidemiological data reveal a striking five-fold increase in the incidence of ICP in twin pregnancies compared to singleton pregnancies, highlighting the substantial risk associated with multiple gestations (Gonzalez et al., 1989). Altogether, results from large epidemiological investigations, clinical observations, and basic research support the notion that increased estrogen levels are a significant cause of ICP. Estrogen is a steroid hormone that has a wide range of physiological and pharmacological activities. Recently, estradiol-17β-D-glucuronide inhibited the expression of multidrug resistance-associated protein 2 (MRP2) at both transcriptional and post-transcriptional levels in the capillaries of hepatocytes in experimental cholestasis rats, suggesting a close relationship between genetic factors and hormones in the pathogenesis of ICP (Stieger et al., 2000). Consistent with this, estrogen levels in the human body are ultimately determined by the expression and function of estrogen receptor (ER) genes *ESR1* and *ESR2*, estrogen biosynthesis, and metabolism key enzyme genes (*CYP17A1*, *CYP19A1*, *CYP1A2*, *CYP1B1*, *CYP3A4*, and *COMT*). Therefore, we hypothesized that specific aberrations in these estrogen-related genes or their expressions might influence estrogen levels and trigger ICP. However, despite numerous investigations, the genetic basis of ICP, particularly those associated with estrogen-related gene variants or expression abnormalities, remains under investigation. Existing research on ICP genetics has predominantly investigated two gene categories: hepatobiliary transporters (*ABCB4*, *ABCB11*, and *ABCC2*) (Liu et al., 2021) and nuclear receptors (*LXR*, *FXR*, and *AHR*) (Liu et al., 2022). Notably, the systematic investigation of rare variants in estrogen-related genes and their potential contribution to ICP pathogenesis remain an important knowledge gap that warrants further exploration.

Building on this evidence, we conducted whole-exome sequencing (WES) in a relatively large sporadic cohort (N = 249) to identify potentially pathogenic rare variants in eight estrogen-related genes associated with ICP and to examine their relationship with disease symptoms and clinical manifestations.

## Materials and methods

### Patients and clinical data

Jiangxi province is located in the Yangtze River valley of China, where the incidence of ICP remains relatively high (∼4%). Patients included in this study received obstetric care and gave birth at the Department of Obstetrics, Jiangxi Maternal and Child Health Hospital between 2018 and 2022. This hospital is a Level A tertiary care institution that manages over 20,000 deliveries annually. Inclusion criteria were based on defining ICP through pruritus and elevated fasting TSBA concentrations (≥10 μmol/L), with or without elevated liver aminotransferase concentrations. All pregnancies complicated by chromosomal abnormalities, those with definitive itching-causing diseases, and patients with a history of hepatobiliary diseases, such as gallstones, autoimmune liver diseases, and liver cirrhosis, were excluded from the study. Based on the above screening criteria, we recruited a total of 249 individuals, all diagnosed with ICP. All patients were informed about the objective of this study and provided written informed consent to participate. This study was approved by the Institutional Review Board of Jiangxi Provincial Maternal and Child Health Hospital in China (Approval No. EC-KT-202204).

Meanwhile, data on maternal demographics (i.e., maternal age, weight, body mass index, gravidity, parity, and gestational weeks at diagnosis), obstetric and medical history, hematological parameters (i.e., white blood cell, red blood cell, and platelet counts), biochemical parameters, including liver function indices (such as TSBA, ALT, and AST), lipid indices (such as total cholesterol, triglyceride, and high-density lipoprotein), ion concentrations (K, Na, Cl, Ca, Mg, and P), and pregnancy outcomes (such as mode of delivery, birth weight, and Apgar score) were retrieved from the hospital records of all individuals with ICP. Hematological parameters were determined using a Sysmex-xn-2000 automatic blood cell analyzer (Sysmex Corporation, Japan). Serum biochemical parameters were determined using an AU5800 automatic biochemical analyzer (Beckman Coulter, Inc., United States). Summary statistics for the clinical features of these 249 patients are provided in our previous study (Liu et al., 2022). Additionally, to compare the variant frequencies of estrogen-related genes between patients with ICP and controls, we enrolled 1,237 local healthy controls during the same study period. We incorporated an independent GEO dataset (GEO accession: GSE46157) comprising two control and four ICP placental samples for placental expression profiling.

### Whole-exome sequencing and analysis

WES is an efficient strategy for targeting the coding regions of the human genome, which comprises approximately 2.5% of the total genome, to identify disease-associated genetic loci (Biesecker, 2010). By leveraging whole-exome sequencing technology, researchers can facilitate the discovery and validation of genetic variants, particularly rare (minor allele frequency, MAF <0.01) and low-frequency (0.01 ≤ MAF ≤ 0.05) variants associated with human disease. This method allows for the detection of many more individuals at a significantly reduced cost and time compared to sequencing the entire genome. Previously, we performed whole-exome sequencing to identify a new ICP susceptibility gene, *ANO8*, and its pathogenic variants (Liu et al., 2020). In addition, we identified novel functional rare variants in ATP-binding cassette (ABC) transporter and receptor genes that are associated with ICP (Lai et al., 2022; Liu et al., 2021; Liu et al., 2022). In this study, DNA was extracted from the peripheral blood of 249 patients with ICP using the Axy Prep Blood Genomic DNA Mini Prep Kit (Item No. 05119KC3, Axygen Scientific, Inc., Union City, CA, United States). The yield, concentration, purity, and integrity of the DNA were evaluated using a NanoDrop spectrophotometer and agarose gel electrophoresis. After quality control, all eligible DNA samples were diluted to a uniform concentration, according to the manufacturer’s protocol, and then subjected to whole-exome sequencing. The most common whole-exome sequencing methods rely on hybridization with oligonucleotide probes to capture targeted DNA fragments, thereby enriching the exonic sequences. In brief, qualified genomic DNA was first randomly fragmented using the Covaris technology, and the resulting fragments were concentrated to a size range of 150–250 bp. Then, the end repair of the prepared DNA fragments was performed, and an “A” base was added at the 3′-end of each strand. Size-selected fragments were amplified, purified, and hybridized using the BGI Exon Kit V4 (BGI, Shenzhen, China) for enrichment. The non-hybridized fragments were washed. The captured products were circularized and amplified by a rolling circle to produce DNA nanoballs. Each resulting library was loaded onto the BGISEQ-500 sequencing platform and subjected to high-throughput sequencing.

We analyzed the whole-exome sequencing data using the pipeline described in our previous study (Liu et al., 2021; Liu et al., 2022). The initial read-filtering process involved three steps: (1) removal of reads contaminated by sequencing adapters; (2) elimination of reads containing >50% low-quality bases; (3) exclusion of reads with >10% unidentified bases, resulting in high-quality clean reads. Clean reads from each sample were mapped to the human reference genome using Burrows–Wheeler Aligner (BWA) software (Li and Durbin, 2009). Variant calling and filtering were performed using the Genome Analysis Toolkit (GATK) (McKenna et al., 2010). After identifying high-confidence variants, the ANNOVAR tool was used to perform variant annotations and predictions (Wang et al., 2010). The final variants and annotation results were used in subsequent analyses. First, we primarily focused on functional variants, including missense, nonsense, splicing, and loss- or gain-of-function of estrogen-related genes. Second, the variants with MAF >0.05 in the 1,237 individuals from the local population and three public databases, including 1000G_ALL, ExAC, and dbSNP, were removed. We particularly concentrated on novel low-frequency or rare functional variants that were predicted to be damaging according to the prediction results of the PolyPhen2, SIFT, and Mutation Taster tools.

### Sanger sequencing

Primer Premier software (version 5.0) was used to design three pairs of primers for three novel variant loci (rs1, rs2, and rs3) in *CYP17A1* to validate the results of exome sequencing. The optimal annealing temperature, amplicon (bp), forward primer, and reverse primer sequences are listed in Table 1.

**TABLE 1:** Primers used for validation of three novel variants in the *CYP17A1* gene.

Patient	Annealing temperature (°C)	Amplicon (bp)	Forward primer (5′–3′)	Reverse primer (5′–3′)
ICP58	52	211	GTG​GCT​CTC​TTG​CTG​CTT​AC	TCA​CTG​TAG​TCT​TGG​TGC​CC
ICP182	52	184	ACA​CGG​CCA​TAT​GCA​TAA​CA	GAG​GAG​ATG​GGC​ACC​ACT​TA
ICP218	52	229	TTG​CTT​CTC​CTG​GGC​TTA​CA	CAC​ACC​TGG​AGT​CAA​CGT​TG

### Evolutionary conservation analysis

We also performed a conservation analysis of the amino acids encoded by the three novel variants, which was verified by Sanger sequencing in vertebrates using the Ensembl Genome Browser.

### Protein structure modeling

As three novel *CYP17A1* missense variants (rs1, rs2, and rs3) were consistently predicted to be damaging by the PolyPhen-2, SIFT, and MutationTaster algorithms, we modeled the structural consequences of each variant. For each variant, we generated mutant protein structures containing single-amino acid substitutions and compared them with the wild-type structure. Subsequently, we first submitted the pre- and post-mutated target amino acid sequences to the SWISS-MODEL repository database (https://swissmodel.expasy.org/, accessed 2024.10.26) to establish a suitable structural template. The UCSF Chimera 1.16 package (https://www.rbvi.ucsf.edu/chimera/docs/credits.html, accessed 2024.10.26), which is a tool for integrated sequence-structure analysis, was used to compare protein structures (Pettersen et al., 2004).

## Results

### Whole-exome sequencing results of the variants in eight estrogen-related genes in 249 individuals with intrahepatic cholestasis of pregnancy

**FIGURE 1:**
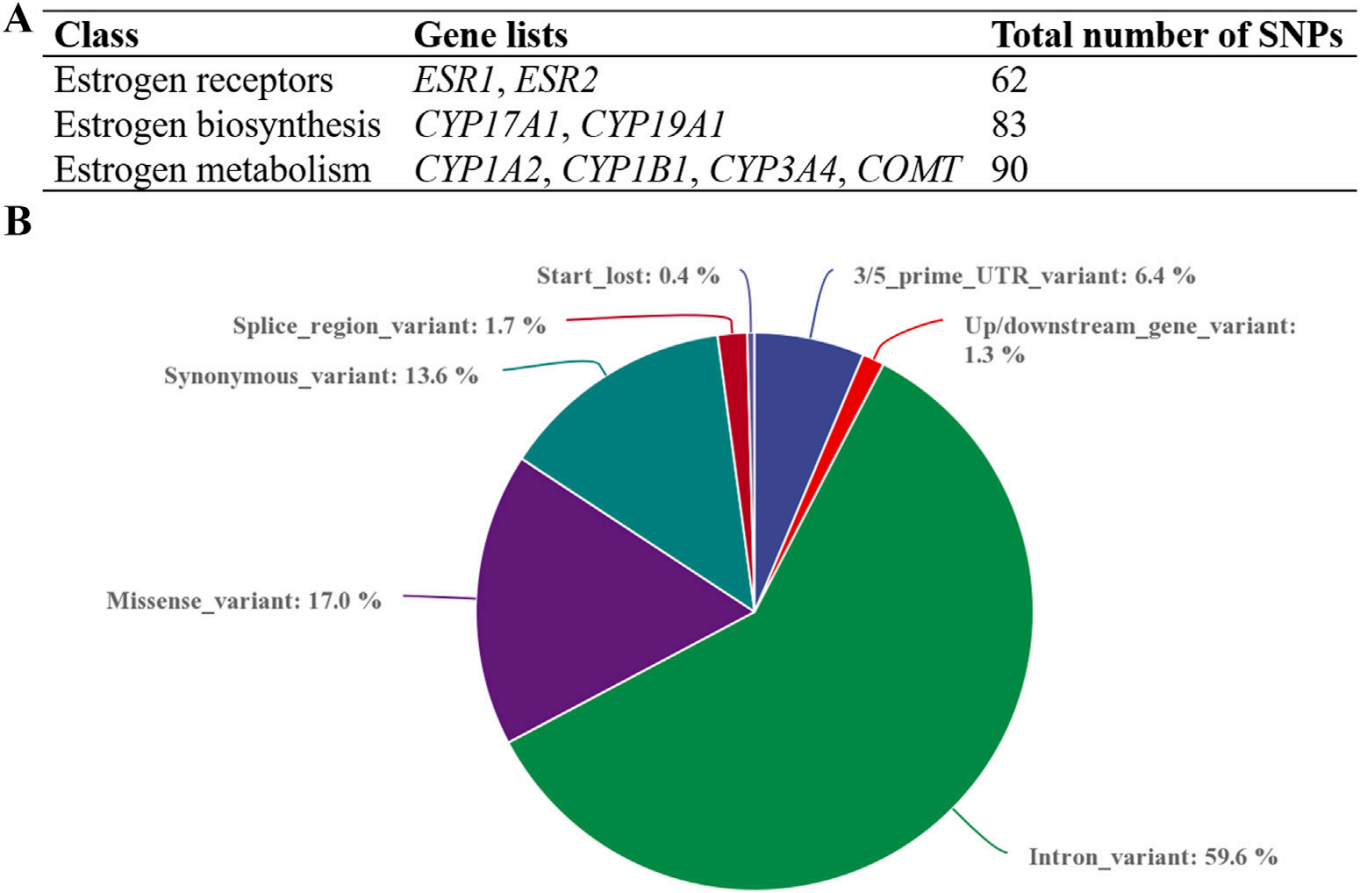
The distribution and numbers of genes and genetic variants from whole-exome sequencing data for ICP. **(A)** The total number of genes and identified SNPs in estrogen-related genes are summarized. **(B)** The percentage of the types of genetic variants in the three series genes.

Whole-exome sequencing identified 235 single-nucleotide polymorphisms (SNPs) across eight estrogen-related genes, including 62 in estrogen receptors, 83 in estrogen biosynthesis, and 90 in estrogen metabolism-associated genes (Figure 1A). The distribution of these variants is presented in a pie chart (Figure 1B), showing that intronic variants accounted for the highest proportion (59.6%), followed by missense variants (17.0%) and synonymous variants (13.6%). Notably, all 249 individuals carried mutations, with the number of variants per individual ranging from 14 to 68.

### Assessing the potential functional impact of the rare novel *CYP17A1* variants

We prioritized paying attention to rare (MAF<0.01) variants in local controls and three public databases, along with potentially damaging variants that are likely to have functional effects. After quality control, five missense variants were identified, namely, rs1339881550 in *ESR2*, rs773366123 in *CYP1A2*, and three novel variants (rs1, rs2, and rs3) in *CYP17A1* (Table 2). These variants were absent in the local population and the three databases. All five variants were assessed using Polyphen2 and SIFT and were predicted to be damaging. In addition, MutationTaster (https://www.mutationtaster.org/, accessed 2024.11.28) predicted all five variants as disease-causing. The missense variant rs1339881550 in the *ESR2* gene was found in a patient with ICP having a bile acid concentration of 17.9 μmol/L. The variant rs773366123 was found in a 32-year-old woman with ICP and pruritus. Women with the variant rs773366123 had a history of ICP and preterm birth. Three novel mutations were identified in the *CYP17A1* gene. The three patients with *CYP17A1* variants did not carry the potential effect loci of known functional genes (such as *ABCB4*, *ABCB11*, and *NR1H4*) for ICP disease, implying that ICP cases with *CYP17A1* variants are not caused by these variants of functionally known genes. The patients (ICP58, ICP182, and ICP218) with these three variants conceived naturally and did not have complications from other pregnancy disorders, such as pregnancy-induced hypertension and gestational diabetes. The patients were 34, 20, and 32 years old, with TSBA levels of 16.3, 26.6, and 56.7 μmol/L, respectively (Table 3). The triglyceride levels of the three individuals were 5.94, 3.26, and 3.85 mmol/L, respectively, all exceeding the maximum reference value of 1.69 mmol/L. Disease onset occurred at 38 weeks +4 days, 37 weeks +3 days, and 33 weeks +1 day of gestation, with delivery at 38 weeks +6 days, 38 weeks +2 days, and 40 weeks, respectively. Birth weights were 3.75, 2.85, and 3.75 kg. Patient ICP218 had a history of ICP, whereas both ICP58 and ICP182 underwent Cesarean delivery during the current pregnancy.

**TABLE 2:** Pathogenic prediction for five missense variants of the *ESR2*, *CYP1A2*, and *CYP17A1* genes in 249 Han Chinese people with intrahepatic cholestasis of pregnancy.

Gene	Rs#	Chr	Position	Allele	Protein change	PolyPhen2	SIFT	MutationTaster	MAF in cases/controls
*ESR2*	rs1339881550	14	64723998	C/T	Arg346His	1.0(D)	0.0(D)	1(D)	2.008E-03/0
*CYP1A2*	rs773366123	15	75042372	T/A	Leu98Gln	1.0(D)	0.0(D)	1(D)	2.008E-03/0
*CYP17A1*	rs1 (Novel)	10	104597037	G/T	Pro28Thr	0.985(D)	0.003(D)	0.99(D)	2.008E-03/0
*CYP17A1*	rs2 (Novel)	10	104596842	A/G	Phe93Leu	1.0(D)	0.0(D)	1(D)	2.008E-03/0
*CYP17A1*	rs3 (Novel)	10	104592367	C/A	Arg347Leu	0.987(D)	0.001(D)	0.99(D)	2.008E-03/0

**TABLE 3:** Descriptive statistics of 33 clinical features of patients ICP58, ICP182, and ICP218 with *CYP17A1* variants.

Feature	ICP58	ICP182	ICP218
Basic information			
Age (years)	34	20	31
BMI (kg/m^2^)	30.27	25.64	28.52
Disease onset (weeks)	38 + 4	37 + 3	33 + 1
Gestational age (weeks)	38 + 6	38 + 2	40
Gravidity (times)	2	1	3
Parity (times)	1	0	1
Hematological parameter			
White blood cell counts (×10^9^)	7.58	8.58	13.07
Red blood cell counts (×10^9^)	3.87	3.9	4.33
Platelets (×10^9^)	93	211	118
Red blood cell distribution width. SD (fL)	49.2	42.6	43.8
Biochemical parameter			
Total serum bile acids (μmol/L)	16.3	26.6	56.7
Alanine aminotransferase (U/L)	7	8	5
Aspartate transaminase (U/L)	13	12	12
γ-glutamyl transpeptidase (U/L)	7	6	6
Alkaline phosphatase (U/L)	112	123	100
Total bilirubin (μmol/L)	11.9	7.7	8
Direct bilirubin (μmol/L)	3.2	3.4	3.1
Indirect bilirubin (μmol/L)	8.7	4.3	4.9
Total cholesterol (mmol/L)	3.75	5.5	5.2
Triglyceride (mmol/L)	5.94	3.26	3.85
High-density lipoprotein (mmol/L)	0.92	1.67	2.41
Low-density lipoprotein (mmol/L)	0.13	2.35	1.02
Uric acid (μmol/L)	300	220	292
K (mmol/L)	3.9	3.3	3.7
Na (mmol/L)	143	138	135
Cl (mmol/L)	108	103	104
Ca (mmol/L)	2.31	2.1	2.6
Mg (mmol/L)	0.82	0.8	0.8
P (mmol/L)	1.31	1	1.1
Outcomes of pregnant women and newborns			
Delivery mode	Cesarean	Cesarean	Vaginal
Birth weight (kg)	3.75	2.85	3.75
Apgar score	9	10	10
Bleeding amount (mL)	300	300	240

### Bioinformatics analysis of three novel variants in the *CYP17A1* gene

**FIGURE 2:**
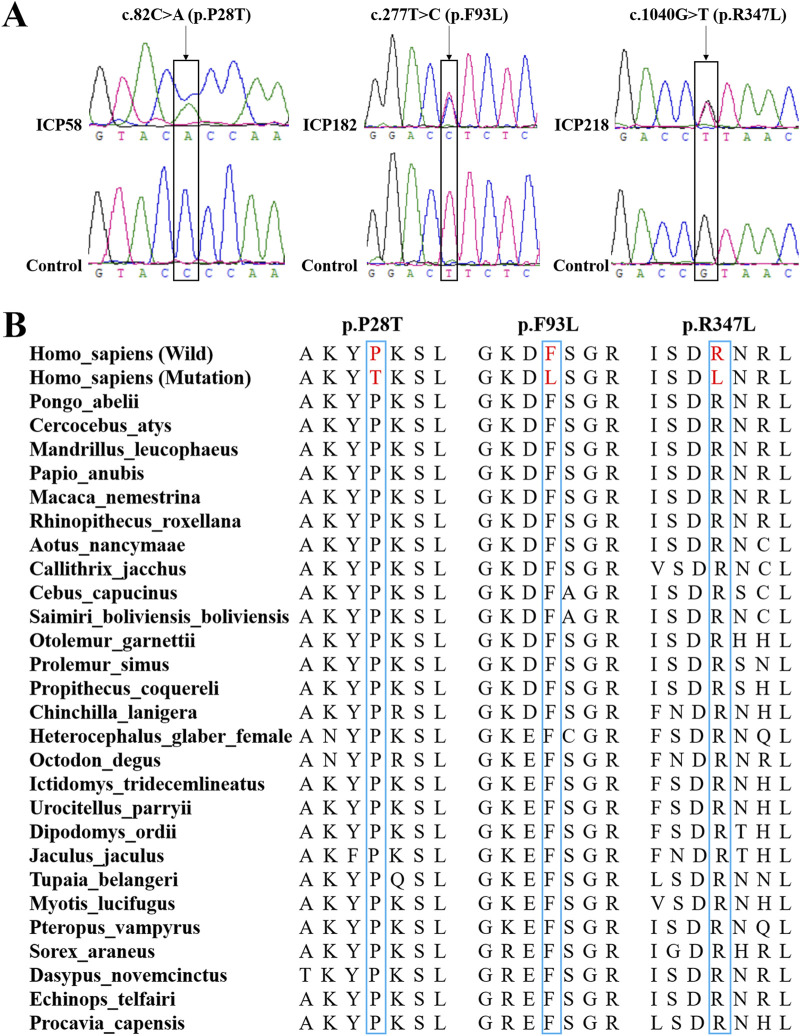
Bioinformatics analysis of the three novel variants of the *CYP17A1* gene. **(A)** Sanger sequencing to confirm these three variants in the CYP17A1 gene. **(B)** Evolutionary conservation analysis of amino acids encoded by these three novel variants.

Using whole-exome sequencing of 249 ICP cases, we identified three novel rare missense variants in *CYP17A1*, each occurring in a distinct individual. Sanger sequencing was subsequently performed to validate the three specific variants in their respective carriers, which confirmed their genotypes (Figure 2A). Evolutionary conservation analysis further revealed that the amino acids at these three variant loci were highly conserved across vertebrate species (Figure 2B).

### Comparison of the protein structural model of the *CYP17A1* p.Pro28Thr, p.Phe93Leu, and p.Arg347Leu variants

**FIGURE 3:**
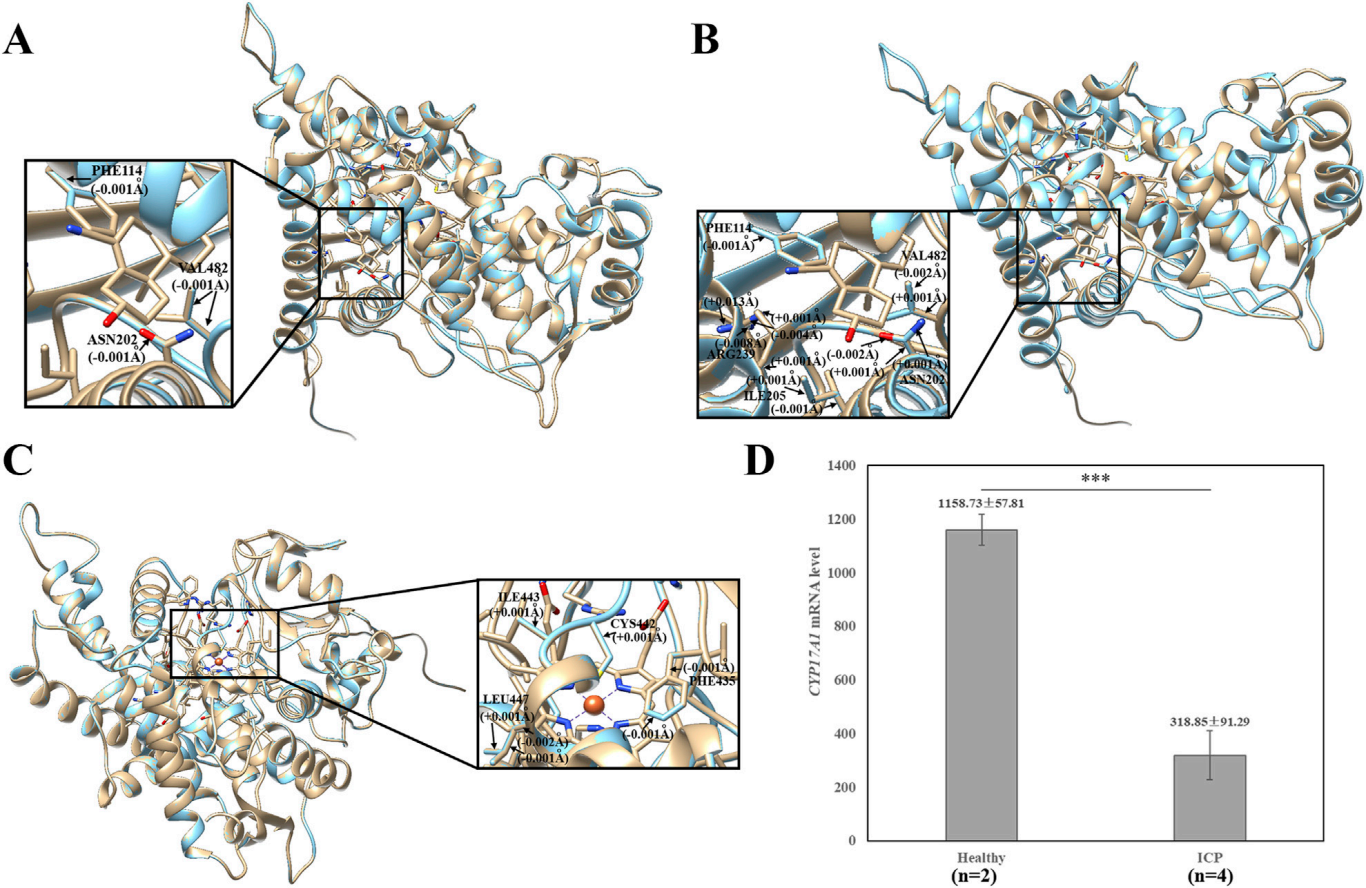
Effects of the *CYP17A1* p.Pro28Thr, p.Phe93Leu, and p.Arg347Leu variants on the protein structure. **(A)** Effects of p.Pro28Thr, **(B)** p.Phe93Leu, and **(C)** p.Arg347Leu on the protein structure. The three-dimensional models of reference and modified (*p.Pro28Thr*, *p.Phe93Leu*, and *p.Arg347Leu*) *CYP17A1* showed gold and blue rounded structures, respectively. The enlarged portion exhibited that the p.Phe93Leu variant has a small change in the chemical bond lengths of amino acid side chains within the steroid-binding and redox partner interaction domain. p.Pro28Thr and p.Arg347Leu variants showed that the steroid-binding and redox partner interaction regions have small changes in the chemical bond lengths, respectively. **(D)** Comparison of the expression level of the CYP17A1 gene between two healthy pregnancy women and four ICP patients.

rs1, rs2, and rs3 novel variants of the *CYP17A1* gene cause a change in the amino acid from proline to threonine, phenylalanine to leucine, and arginine to leucine at positions 28 (Pro28Thr), 93 (Phe93Leu), and 347 (Arg347Leu), respectively. Consistent with this, they were located in the membrane attachment domain, steroid-binding domain, and redox partner interaction domain. To further investigate the possible effects of the three missense variants on the protein structure, we simultaneously used the UCSF Chimera 1.16 package to compare the *CYP17A1* reference protein structure and the protein structure of the variants, including p.Pro28Thr, p.Phe93Leu, and p.Arg347Leu. For the three variants, there were no visible changes in the overlaid protein structures (Figures 3A–C). Structural analysis revealed subtle alterations in the chemical bonding patterns within the three major functional domains. For the variant p.Phe93Leu, compared with the reference protein structure, the mutation caused a slight change in the chemical bond lengths of amino acid side chains within the steroid-binding and redox partner interaction domains at positions Phe114, Asn202, Ile205, Arg239, and Val482 (Figure 3B). Similarly, the other two novel missense variants, p.Pro28Thr and p.Arg347Leu, exhibited altered chemical bonding patterns at specific residue positions: p.Pro28Thr: 114, 202, and 482 (Figure 3A); p.Arg347Leu: Phe435, Cys442, Ile443, and Leu447 (Figure 3C). These variants in various domains may cause changes in the substrate-binding state and activities of 17α-hydroxylase and 17, 20-lyase.

To further explore possible biological functions, we investigated the biological process and tissue expression of *CYP17A1* using the HumanBase (http://hb.flatironinstitute.org). The main biological processes involving CYP17A1 include drug metabolism, regulation of hormone levels, cellular ketone metabolism, and steroid metabolism. In addition, tissue expression results showed that the top nine tissues with the highest CYP17A1 expression were the testis (confidence: 0.49), blood (0.39), fetus (0.38), liver (0.35), ovary (0.33), nervous system (0.32), brain (0.31), mammary glands (0.31), and placenta (0.30). To examine the genetic basis of *CYP17A1* in ICP, we compared its mRNA expression levels in placental tissue between healthy controls (*n* = 2) and patients with ICP (*n* = 4) using GEO datasets (Accession No: GSE46157) reported by Du et al. (2014). The ICP group demonstrated significantly lower *CYP17A1* mRNA expression levels (318.85 ± 91.29) than the healthy controls (1,158.73 ± 57.81; *p* = 0.00032) (Figure 3D).

## Discussion

Numerous studies have established associations between abnormal estrogen levels and various obstetric and gynecological disorders, including endometrial cancer, ovarian cancer, uterine fibroids, endometriosis, recurrent miscarriage, and ICP (Itsekson et al., 2007; Kim et al., 2013; Reyes and Simon, 1993; Xiao et al., 2021). In view of these observations, we suggest that genes related to the physiological effects of estrogen may be considered candidates for ICP. Therefore, we selected eight genes (estrogen receptor genes: *ESR1* and *ESR2*; estrogen biosynthesis genes: *CYP17A1* and *CYP19A1*; and estrogen metabolism genes: *CYP1A2*, *CYP1B1*, *CYP3A4*, and *COMT*) associated with estrogen levels for whole-exome sequencing of 249 ICP samples to investigate possible candidate pathogenic genetic loci.

The ER is a member of the ligand-dependent transcription factor superfamily (steroid hormone, vitamin D3, and retinoic acid receptors) and has transcription factor properties (Hart and Davie, 2002). There are two subtypes of ER: ERα and ERβ, which are encoded by the *ESR1* and *ESR2* genes, respectively (Lazari et al., 2009). Estrogen regulates the expression of a series of genes by binding to ERs in target organs and thus plays an important physiological role. Previous studies have confirmed an association between ER polymorphisms and endometrial cancer, endometriosis, and polycystic ovary syndrome (Kim et al., 2010; Mear et al., 2020; Weiderpass et al., 2000). In the context of ICP, it has been suggested that alterations in ER-mediated synthesis of low-density lipoprotein receptors and alanine transporters, along with decreased synthesis of organic anion and cholic acid transporters in hepatocytes, are related to the development of cholestasis (Marino et al., 2001). Multiple studies have demonstrated significantly elevated levels of serum-free estriol and placental ER expression in patients with ICP compared to healthy pregnant controls (Fang and Fang, 2022; Feng et al., 2022). Moreover, to the best of our knowledge, only two studies have reported associations between ICP and *ESR1* intron 1 polymorphisms (XbaI A/G substitution and Pvu II T/C substitution) (Eloranta et al., 2002). The results from these two studies suggest that there is no correlation between the two *ESR1* variants and ICP. In the present study, we did not identify any of these variants in a cohort of 249 individuals. A reasonable explanation for this finding may be genetic heterogeneity within the population or the possibility that these two intronic variants are located far from exons, making them undetectable using the methods proposed in this study. For *ESR2*, only one study by Zhang et al. (2006b), conducted in Chengdu, China, has investigated two specific polymorphisms, the Rsa I variant in exon 5 and the Alu I variant in exon 8, for their potential association with ICP. They found that the Alu I polymorphism in exon 8 of *ESR2* may be associated with susceptibility to ICP. Both variants were identified in our cohort of 249 individuals, with minor allele frequencies (MAFs) of 0.33 and 0.13, respectively. These factors were not associated with the ICP risk in Jiangxi, which could be attributed to regional differences.

Estrogen metabolism is catalyzed by a series of key CYP enzymes, such as *CYP1A2*, *CYP1B1*, *CYP3A4*, and catechol-O-methyltransferase *COMT*. Previous case–control studies, which included 100 patients with ICP and 100 healthy pregnant women as controls, showed that the variant rs1056827 (c.355G>T, p.Ala119Ser) may be associated with the risk of ICP. This variant was also detected in our cohort with a frequency of 0.35 (87/249). The MAFs of this variant in 1000G_ALL and ExAC were 0.36 and 0.41, respectively. Using Fisher’s test, the differences in the frequencies of this variant between 249 cases and the two public databases (1000G_ALL and ExAC) were both significant (*p* = 3.30e^−09^; 4.62e^−13^). Therefore, our results further support the hypothesis that rs1056827 is associated with the risk of ICP. Nevertheless, whether this locus contributes to ICP pathogenesis requires confirmation through functional studies in cells and animal models in future research.

Human *CYP17A1* encodes a single microsomal enzyme, P450c17, with two distinct activities: 17α-hydroxylase and 17,20-lyase (Van Den Akker et al., 2002). *CYP17A1* is located on chromosome 10, spans 6,569 bp, and consists of eight exons. Considering that CYP17A1 encodes the key enzyme involved in the rate-limiting step of estrogen biosynthesis, its variation is regarded as an early signal of the upregulation of estrogen production and metabolism. These alterations may influence the occurrence and progression of many estrogen-dependent diseases and are considered potential pathogenic factors in hormone-dependent tumors and other conditions. Many previous studies have linked *CYP17* polymorphisms to the risk of breast cancer, endometrial cancer, ovarian cancer, uterine fibroids, endometriosis, recurrent miscarriage, and ICP (He et al., 2022; Zhang et al., 2006a). At present, most studies on *CYP17A1* focus on the T→C SNP in the 34 bp (promoter region) upstream of its 5′ transcription start site, which forms a new SP1 promoter site (CCACC box) (Carey et al., 1994). Because the number of promoter elements is related to the promoter activity, the transcriptional capacity of polymorphic alleles may be enhanced, leading to increased estrogen production. Found that the C allele alters endogenous hormone levels. Compared to women with the TT genotype, those with the CC genotype had elevated levels of estrone (E1, +14.3%, *p* = 0.01) and estradiol (E2, +13.8%, *p* = 0.08). In addition, also confirmed that the C mutation allele causes the accumulation of more estrogen in the serum of premenopausal women. Consistent with this, our current study also found the −34T>C variant in 218 of 249 patients with ICP (87.55%, 218/249). The MAFs of the −34T>C variant in the 1000G_ALL and 1000G_ExAC were 0.41 and 0.40, respectively. We found no significant difference (*p* = 0.53) in the frequency of this variant between 249 cases and the 1000G_ALL database. The non-significant reason might be the limited number of samples involved or the fact that the −34 T>C variant was not associated with ICP.

In addition to the −34T>C variant, three novel variants were also identified: p.Pro28Thr, p.Phe93Leu, and p.Arg347Leu. Variants in enzymes are more likely to affect their content or catalytic activity, thus affecting the relevant biological pathways. A study by Di Cerbo et al. (2002) showed that the p.Phe93Cys-mutated CYP17 retains only 10% of both 17α-hydroxylase and 17,20-lyase activities. In addition, Van Den Akker et al. (2002) demonstrated, through clinical observations and *in vitro* expression studies, that Phe114 and Asp116 variants in the steroid-binding domain resulted in combined 17α-hydroxylase and 17,20-lyase deficiency, while the p.Arg347Cys and p.Arg347His variants in the redox partner interaction domain led to isolated 17,20-lyase deficiency. Therefore, based on the observed structural changes associated with the p.Pro28Thr, p.Phe93Leu, and p.Arg347Leu variants, we speculated that p.Phe93Leu is most likely to impair both 17α-hydroxylase and 17,20-lyase activities, whereas the p.Arg347Leu variant may result in 17,20-lyase deficiency. Notably, the pattern of enzymatic alteration differs between monoallelic and biallelic mutations depending on their genetic and functional characteristics, as exemplified by *G6PC*, which requires biallelic mutations to affect its activity (Lei et al., 1993), and single *FGFR3* mutations that cause achondroplasia through abnormal enzymatic activity (Rousseau et al., 1994). Therefore, the effects of these three new variants on enzymatic activity need to be functionally validated by constructing wild-type and mutant plasmids and transfecting the cells.

One of the key strengths of this study was that the 249 patients with cholestasis were all from a tertiary care hospital, where a consistent definition of the disease was used to include TSBA in the diagnostic criteria. Importantly, detailed clinical data and documentation of disease characteristics in all individuals provide potential clues for further understanding of the mechanisms by which genetic loci contribute to ICP pathogenesis. However, the variants found based on the population in Jiangxi Province may be limited in the scope of application. Therefore, it is necessary to integrate data from multiple studies and centers in the future.

## Conclusion

This study identified five potentially pathogenic missense variants in estrogen-related genes through whole-exome sequencing of 249 patients with ICP, including three novel *CYP17A1* variants (p.Pro28Thr, p.Phe93Leu, and p.Arg347Leu) that were predicted to impair enzyme function. These findings expand our understanding of the genetic architecture of ICP and highlight the role of dysregulation of the estrogen pathway in disease pathogenesis. Although further functional studies are required to validate their clinical significance, these variants may serve as potential biomarkers for ICP risk assessment. Overall, these results underscore the importance of genetic screening in high-risk populations to improve ICP diagnosis and management.

## Data Availability

The original contributions presented in the study are included in the article/supplementary material; further inquiries can be directed to the corresponding authors.
